# Neutralizing Antibody-Mediated Protection from Prior Delta Variant Infection Against Omicron BA.5 Sub-Lineage Reinfection One Year Later: A Prospective Cohort Study

**DOI:** 10.3390/vaccines12111211

**Published:** 2024-10-25

**Authors:** Shihan Zhang, Yin Wang, Guo Xu, Chen Dong, Hua Tian, Chuchu Li, Xiaoxiao Kong, Jiefu Peng, Haodi Huang, Aidibai Simayi, Fengcai Zhu, Jianli Hu, Ke Xu, Changjun Bao, Hui Jin, Liguo Zhu

**Affiliations:** 1 Department of Epidemiology and Health Statistics, School of Public Health, Southeast University, Nanjing 210009, China; shihanzhang.1997@gmail.com (S.Z.);; 2 Key Laboratory of Environmental Medicine Engineering, Ministry of Education, School of Public Health, Southeast University, Nanjing 210009, China; 3 Department of Acute Infectious Disease Control and Prevention, Yangzhou Center for Disease Control and Prevention, Yangzhou 225007, China; 4 Key Laboratory of Infectious Diseases, School of Public Health, Nanjing Medical University, Nanjing 211166, China; 5 Department of Acute Infectious Disease Control and Prevention, Jiangsu Provincial Center for Disease Control and Prevention, Nanjing 210009, China; 6 National Health Commission (NHC) Key Laboratory of Enteric Pathogenic Microbiology, Jiangsu Provincial Center for Disease Control and Prevention, Nanjing 210009, China; 7 Jiangsu Province Engineering Research Center of Health Emergency, Nanjing 210009, China; 8 Jiangsu Provincial Medical Key Laboratory of Pathogenic Microbiology in Emerging Major Infectious Diseases, Nanjing 210009, China

**Keywords:** SARS-CoV-2, Delta variant, Omicron BA.5, reinfection, neutralizing antibody

## Abstract

Background: Previous SARS-CoV-2 infection provides some level of protection against reinfection. However, few studies have evaluated the neutralizing antibody (NAb) response after Delta variant infection and its ability to prevent reinfection with Omicron BA.5 one year later. Methods: This prospective cohort study included 431 patients who recovered from Delta variant infection. We measured their serum NAb titers against both Delta and Omicron BA.5 using microneutralization tests. Results: Over a 17-month follow-up, 17.9% of the participants were reinfected with Omicron BA.5. Younger adults (18–65 years) and individuals who did not receive booster immunization had significantly higher reinfection rates than older adults (>65 years) and those who received boosters (*p* < 0.05). Notably, reinfection rates were higher in post-vaccination breakthrough cases than in individuals who were naturally infected. However, booster immunization reduced reinfection rates within the breakthrough group. We found no significant association between Delta NAb levels and protection against Omicron BA.5 reinfection (*p* > 0.05). Cross-neutralization assays showed a 7.1-fold reduction in NAb efficacy against Omicron BA.5. Conclusions: Delta-variant infection-induced NAbs did not strongly predict protection against Omicron BA.5 reinfection. However, booster immunization effectively reduced the reinfection rate approximately one year after the initial Delta infection.

## 1. Introduction

Coronavirus disease 2019 (COVID-19) remains a significant public health burden owing to the existence and mutation of severe acute respiratory syndrome coronavirus 2 (SARS-CoV-2). Omicron BA.4 and BA.5 were first identified in South Africa in January 2022 [1], and the first domestic outbreak of Omicron BA.5 was reported in China in July 2022. Owing to multiple mutations in its spike protein, such as L452R and F486V, which enhance its transmissibility [2], the Omicron BA.5 subvariant has progressively replaced the earlier Omicron BA.1 and BA.2 subvariants [3].

Previous COVID-19 infection was shown to provide protection against reinfection and symptomatic disease caused by the predecessors of Omicron: Alpha (B.1.1.7), Beta (B.1.351), and Delta (B.1.617.2) variants [4,5]. Before the emergence of the Omicron variants, a previous infection reduced the risk of reinfection by 87% [6]. However, prior infection was less protective against reinfection with Omicron overall [7,8]. The Omicron BA.5 subvariant has also demonstrated a strong ability to evade neutralizing antibodies [9]. Vaccination, infection with SARS-CoV-2, and a combination of both provide varying degrees of protection [10]. However, both vaccine-induced and infection-induced immunity will wane over time [11]. Yang et al. recently reported that vaccine efficacy against symptomatic infection waned over time, with an average decrease of 13.6% per month, but it can be enhanced by a booster [12].

According to a report from the China CDC, as of 20 April 2023, the predominant circulating strains are BA.5.2 and its sub-lineages (accounting for 66.2%) and BF.7 and its sub-lineages (accounting for 29.8%). There is limited information on Omicron BA.5 sub-lineage reinfections among individuals who were initially infected with the Delta variant, both domestically and abroad, and there is almost no research on the relationship between serum-neutralizing antibody levels and reinfection. Therefore, in this study, we conducted a follow-up investigation for over one year on individuals who were initially infected with the Delta variant in Yangzhou City, Jiangsu Province, in July 2021 to investigate whether they experienced reinfection during the Omicron BA.5 sub-lineage epidemic from 5 December 2022 to 15 January 2023. We also explored the ability of serum-neutralizing antibody levels against Omicron BA.5 sub-lineages to prevent reinfection and identified factors that affect reinfection, providing preliminary answers regarding Omicron BA.5 sub-lineage reinfections among individuals who were initially infected with the Delta variant, as well as feasible measures to reduce the reinfection rate.

## 2. Methods

### 2.1. Study Design

We conducted a prospective cohort study in Yangzhou City, Jiangsu Province, China. This study was approved by the Ethics Committee of the Jiangsu Provincial Center for Disease Prevention and Control (JSJK2021-B013-01).

### 2.2. Study Population and Serum Sampling

The study cohort included 431 patients initially infected with the SARS-CoV-2 Delta strain in Yangzhou in July 2021, who were followed up and called back to check reinfection status during the Omicron BA.5 sub-lineage pandemic from 5 December 2022 to 15 January 2023. During a follow-up period of up to one and a half years, we rigorously monitored the symptoms of the study participants. If any symptoms of upper respiratory tract infection were observed, such as fever, local healthcare research institutions promptly conducted nucleic acid testing to identify whether a COVID-19 infection had occurred. Therefore, as a cohort study, through symptom monitoring and nucleic acid testing, we ensured the accuracy and reliability of outcome event occurrences. The enrolled subjects were divided into two categories according to vaccination status before initial infection: natural infection and breakthrough infection. Unvaccinated infection was defined as patients receiving no COVID-19 vaccine doses. A breakthrough infection was defined as detecting SARS-CoV-2 on an RT-PCR assay performed 14 or more days after vaccination with a second dose of CoronaVac/BBIBP/ZF2001. Serum was collected from 431 patients about one month into the convalescent period when antibody levels were more stable than in the acute period. The clinical data included COVID-19 immunization history after initial infection, demographics, and acute-phase disease severity classification at the time of initial infection. According to the Diagnosis and Treatment Protocol for Novel Coronavirus Pneumonia [13], the clinical spectrum of COVID-19 ranges from mild to severe/critical. COVID-19 patients with mild clinical symptoms but without pneumonia manifestation found by imaging and with typical clinical symptoms (fever, respiratory tract symptoms, etc.) and pneumonia manifestation found by imaging were classified as mild and normal cases, respectively. Severe/critical COVID-19 patients met any of the following criteria: (i) respiratory distress, respiratory rates of ≥30 breaths/min; (ii) percutaneous oxygen saturation (SpO_2_) of ≤93% at rest; (iii) arterial oxygen tension/inspiratory oxygen fraction ratio (PaO_2_/FiO_2_) of ≤300 mm Hg; (iv) greater than 50% lesion progression within 24 to 48 h in pulmonary imaging; (v) respiratory failure requiring mechanical ventilation; (vi) shock; and (vii) complications from other organ failures requiring monitoring and treatment in the intensive care unit (ICU).

### 2.3. Serum Microneutralization Test

A live-virus MN assay was established for SARS-CoV-2 NAb detection. Two strains of SARS-CoV-2 (Delta and Omicron BA.5) were used as the challenge viruses, and the 50% cell culture infective dose (CCID50)/50 μL was calculated with Kärber’s formula [14]. Briefly, the serum samples were inactivated at 56 °C for 30 min and then diluted serially twofold, starting at a dilution of 1:8 and ending at 1:1024. In total, 50 μL of each diluted serum was mixed with an equal volume of challenge virus containing approximately 100 CCID_50_/50 μL. After neutralization at 36.5 °C for 2 h in a 5% CO_2_ incubator, a Vero-E6 cell suspension was added to the plate with the maintenance medium to form a cell monolayer. The plate was kept at 36.5 °C for 5 days in the 5% CO_2_ incubator. During this period, the cytopathic effect (CPE) was observed daily. The titer of the NAbs was calculated using the Reed–Muench method [15]. Serum with an NAb titer of greater than or equal to 1:8 was considered positive. To calculate geometric mean concentration (GMC), antibody titers of <1:8 and >1:1024 were assigned as 1:4 and 1:1024, respectively. The national standard for SARS-CoV-2 NAbs (No.280034-202001) assigns a concentration of 1000 units per mL, available from the China National Institute for Food and Drug Control and was used to convert the neutralizing titer of the serum to U/mL in this study. A detailed description can be found in our team’s previous research [16].

### 2.4. Chemiluminescence Immunoassay

We applied an ACE2–RBD-inhibiting antibody Chemiluminescence Immunoassay Kit (Bioscience Co., Chongqing, China) to detect serum-neutralizing antibodies in individuals reinfected with the Omicron BA.5 variant. The assay was performed according to the manufacturer’s protocol, and serum NAb levels were calculated based on the ACE2–RBD-inhibiting antibody concentration. An automated magnetic chemiluminescence analyzer Axceed 260 (Bioscience Co.) was used for signal detection, with relative light units (RLUs) measured using the instrument’s optical system. The chemiluminescent signal was measured as RLUs, and the titers of SARS-CoV-2 ACE2 competitive antibodies in the serum were quantified using the sample/cutoff (S/CO) ratio. An S/CO ratio of ≥1.0 was considered positive, while an S/CO ratio of <1.0 was deemed negative. The detection performance of the kit was reported by the manufacturer, with an 85–90% coincidence rate for neutralization antibody detection [17,18].

### 2.5. Study Outcome

The primary outcome was a positive SARS-CoV-2 RT-PCR or antigen (AG) test during the period of Omicron BA.5 dominance from 5 December 2022 to 15 January 2023. Reinfection in this study is defined as a positive SARS-CoV-2 RT-PCR or AG test occurring ≥ 90 days after initial testing [19]. Through whole-genome sequencing analysis (Illumina, San Diego, CA, USA) [20], all reinfected subjects were found to be infected with the Omicron BA.5 sub-lineage Omicron BA.5.2/BF.7.

### 2.6. Statistical Analysis

We used Student’s *t*-test and the Related-Samples Wilcoxon Signed Rank Test or the Kruskal–Wallis test for normally and non-normally distributed variables, respectively. Results with heavily skewed distributions were normalized via log transformation. Log-transformed NAb titers against different variants were compared using Student’s *t*-test or one-way ANOVA. A two-tailed *p*-value of <0.05 indicated statistical significance throughout the study. Analyses were conducted using R v4.2.2 and GraphPad PRISM 9.4.

## 3. Results

### 3.1. Reinfection with Omicron BA.5.2/BF.7 in Individuals Previously Infected with SARS-CoV-2 Delta

**Figure 1:**
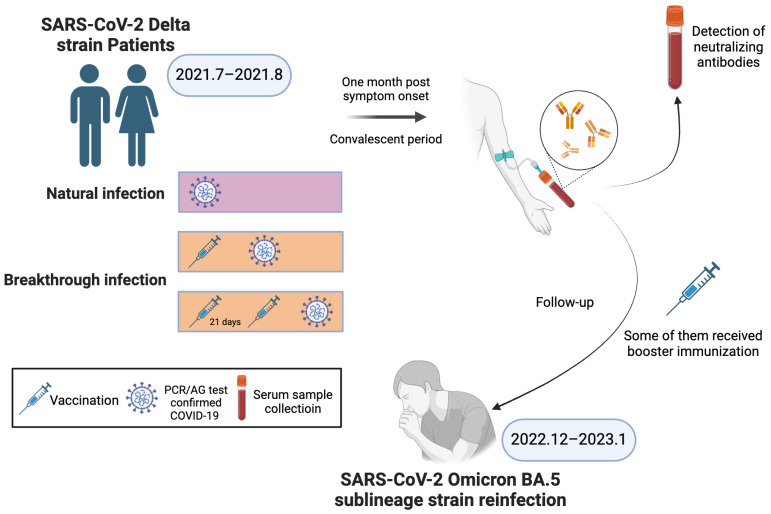
Study scheme. This study employed a cohort design, enrolling individuals who had been infected with the Delta variant. The primary outcome of interest was the incidence of the Omicron BA.5 sub-lineage reinfection observed over a follow-up period of 17 months. The main observational parameter was the neutralizing antibody levels one month into the recovery phase for individuals infected with the Delta variant.

Based on the study design presented in Figure 1, we included 431 individuals with infections in Yangzhou City during the Delta wave outbreak in July 2021 and tracked their reinfection status during the Omicron BA.5 sub-lineage outbreak at the end of 2022. Table 1 shows the reinfection status of different demographic groups. Through follow-up, we found that 77 individuals were reinfected with the Omicron BA.5.2/BF.7 variant strain, resulting in a reinfection rate of 17.9%. Among individuals under 18 years old, five cases of reinfection were observed, resulting in a reinfection rate of 10.9%. In the 18-to-65-year-old age group, 56 cases of reinfection were observed, resulting in a reinfection rate of 22.9%. The reinfection rate for individuals over 65 years old was 11.4%. There was a statistically significant difference in reinfection rates between different age groups (*p* = 0.008). We did not observe any statistically significant differences between different sex and clinical classification groups (*p* > 0.05).

Based on the vaccination status at the time of the initial infection, we categorized the study participants into those with natural infections and those with breakthrough infections. The table shows that the reinfection rate was higher among individuals with breakthrough infections than those with natural infections (21.6% vs. 12.5%, *p* = 0.016). Furthermore, we conducted a stratified analysis of the study participants based on whether they had received booster immunization and the number of booster doses received after the initial infection. Using trend Chi-square analysis, we found that individuals who had not received booster immunization had the highest reinfection rate (24.5%), and the reinfection rate showed a decreasing trend with an increasing number of booster doses (*p* = 0.007). Finally, we also considered the impact of the time interval between the last booster dose and the initial infection on reinfection. We found that as the time interval increased, the reinfection rate showed a decreasing trend, but the inter-group difference was not significant (*p* > 0.05). The booster immunization status of individuals who were naturally infected and individuals with breakthrough infections during the follow-up period is presented in Table S1. This table compares the number of participants who received one, two, or three or more booster doses, as well as the median interval between the last booster dose and the initial infection for each group.

### 3.2. The Association between Neutralizing Antibody Levels During the Convalescent Period of Delta Infection and Omicron Reinfection

Figure S1 compares neutralizing antibody (NAb) levels during the recovery period after Delta infection between individuals who experienced Omicron BA.5 reinfection and those who did not. The geometric mean concentration (GMC) of Delta NAbs during the recovery period in individuals who were reinfected was 9702.6 U/mL (IQR: 6464.1–25,860.4), with a positivity rate of 96.1%. By contrast, for those without reinfection, the GMC was 10,369.2 U/mL (IQR: 5501.8–32,909.4), with a positivity rate of 98.3%. No statistically significant differences were observed between the two groups in terms of Delta NAb levels (*p* = 0.743) or positivity rates (*p* = 0.205). Figure S1 shows that the distribution of Delta NAb levels was similar between the reinfected and non-reinfected groups, suggesting that the convalescent Delta NAb levels were not significantly associated with the risk of subsequent Omicron BA.5 reinfection.

To increase comparability between groups, we stratified the analysis of the relationship between Delta NAb levels during the recovery period and Omicron BA.5 reinfection based on the vaccine status at the time of initial infection (natural infection/breakthrough infection) and whether or not an individual received a booster immunization after initial infection. The relevant results are shown in Tables S2 and S3. We were surprised to find no direct association between neutralizing antibody levels against the Delta strain and Omicron BA.5.2/BF.7 reinfection, even in the population without any SARS-CoV-2 immunization (*p* > 0.05). This suggests that the intuitional role of serum Delta NAb levels is limited in determining the occurrence of Omicron BA.5.2/BF.7 reinfection.

In addition, we stratified the study population based on their Delta NAb levels and compared the reinfection rates between individuals in the upper and lower quartiles of the neutralizing antibodies. The results are shown in Table S4. Among the top 25% of individuals with higher NAb levels, 20 were reinfected, accounting for 16.9% of this group. Among the bottom 25% of individuals with lower NAb levels, 18 were reinfected, accounting for 15.9% of this group, while 84.1% remained uninfected. There was no statistically significant difference in reinfection rates between the two groups (*p* > 0.05). The Delta NAb levels of the study participants can be found in Table S5.

### 3.3. Cross-NAb Response to Omicron BA.5 and Its Association with Reinfection

**Figure 2:**
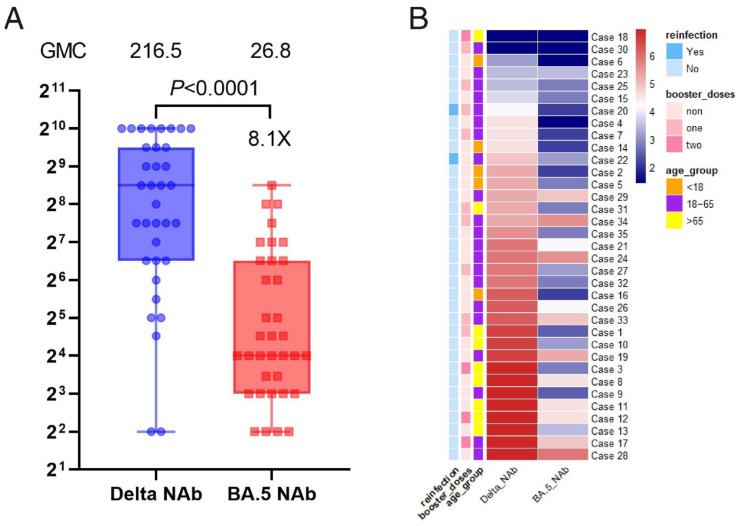
The cross-neutralizing ability of 35 individuals with Delta variant infections against Omicron BA.5. (**A**) Box-and-whisker plot of NAb levels. The Y-axis was scaled in Log2 units. *p*-value was obtained from a two-sided t-test performed on the log-transformed NAb titers. (**B**) Heatmap of Delta NAbs and Omicron BA.5 NAbs for 35 cases. Each square represents an individual, with NAb levels in the logarithmically transformed scale. The color gradient ranges from dark blue, indicating lower antibody levels, to dark red, indicating higher antibody levels. One outcome variable and two significant covariates are displayed in the row annotations of the figure.

We randomly selected 35 serum samples from individuals who had recovered from Delta strain infection and used a cross-neutralization assay to investigate the neutralizing activity against Omicron BA.5. The Delta NAb and Omicron BA.5 NAb levels for each individual are shown in Figure 2A (specific neutralization levels are provided in Table S6). The GMC of the neutralizing antibodies against the Delta variant was 216.5 U/mL in 35 serum samples, while the GMC against Omicron BA.5 was 26.8 U/mL. The latter exhibited a 7.1-fold decrease in average neutralizing ability, and the difference between the two groups was statistically significant (*p* < 0.0001). We further illustrated the changes in neutralizing ability at the individual level using a heatmap (Figure 2B). The main part of the figure shows the NAb levels of the 35 Delta-infected sera against different strains (since Omicron BA.5 NAbs and Delta NAbs were not on the same scale, we transformed the two sets of values using logarithms to facilitate displaying inter-individual differences). In addition, we added row annotations for each case, including an outcome indicator (reinfection) and two important covariates (booster doses and age group).

Figure 2B shows that the Delta NAb levels in the 35 serum samples were generally higher than in the Omicron BA.5 NAb levels. Follow-up showed that two cases had Omicron BA.5 reinfections, one without receiving a booster dose and the other with one booster dose. Both cases were in the 18–65 age group.

### 3.4. The Consistency of NAb Levels in Cases of Reinfection Compared with Initial Infection

**Figure 3:**
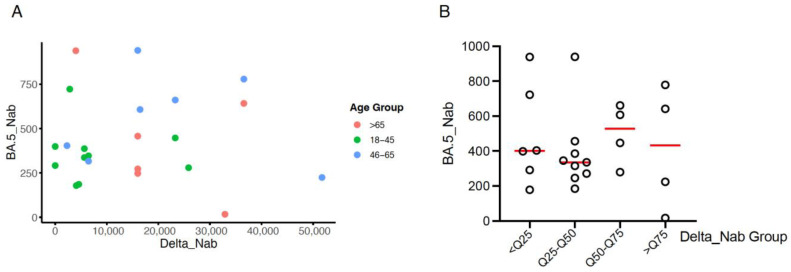
The association between initial Delta variant NAb levels and Omicron BA.5 reinfection NAb levels. (**A**) Scatter plot comparing Delta NAb and Omicron BA.5 NAb levels across different age groups (18 < 45, 46–65, and >65 years). No significant correlation was observed between Delta NAb and Omicron BA.5 NAb levels in any age group. (**B**) Comparison of Omicron BA.5 NAb levels across quartile groups based on Delta NAb levels (<Q25, Q25–Q50, Q50–Q75, and >Q75). The horizontal lines represent the median NAb levels for each group.

From the group of 77 individuals who experienced reinfection with the Omicron BA.5 strain, we collected 28 serum samples based on participant compliance and the needs of the study. We then compared the neutralization levels in reinfection versus initial infection among the 28 individuals, with the results presented in Figure 3. After identifying and removing outliers of neutralization levels using the interquartile range (IQR) method, no significant correlation was observed between Delta NAb levels and Omicron BA.5 NAb levels (Figure 3A). In Figure 3A, we further categorized participants into different age groups (<45, 46–65, and >65 years) to examine the potential age-related differences in the relationship between Delta NAb and Omicron BA.5 NAb levels. However, no clear age-related trend was observed between these two variables. In addition, we divided the Delta NAb levels of initial infections into quartiles (<Q25, Q25–Q50, Q50–Q75, and >Q75) to explore whether there were differences in Omicron BA.5 NAb levels between these groups, as shown in Figure 3B. Using the Kruskal–Wallis test, we found a *p*-value of 0.75, indicating no statistically significant differences in Omicron BA.5 NAb levels between the four quartiles.

## 4. Discussion

In this prospective cohort study, we observed a reinfection rate of 17.9% for individuals who were initially infected with the Delta variant and 17 months later infected with the Omicron BA.5 sub-lineage. Notably, variations were observed in the reinfection rates among different age groups, with post-vaccination breakthrough cases unexpectedly having higher reinfection rates than natural infections. Furthermore, individuals with boosted immunity following primary infection had a lower reinfection rate than those without boosted immunity. Because infection-induced NAbs are a critical component of the immune response to subsequent challenges with SARS-CoV-2 [21], we evaluated NAbs against the Delta strain in the sera of individuals with prior Delta variant infections using a live-virus micro-neutralization assay. Interestingly, we found no direct association between Delta NAbs and protection against reinfection with the Omicron BA.5 sub-lineage.

Our study revealed variations in reinfection rates among different age groups, with the 18–65 age group showing a higher reinfection rate than individuals aged 65 and above. This phenomenon may be due to a larger proportion of older patients developing severe or critical illness, whereas younger patients developed mild or moderate symptoms or were asymptomatic during the prime infection. Previous studies have shown that severe/critical patients gain greater protection against reinfection than mild and moderate cases [22,23].

The higher reinfection rate observed among individuals with breakthrough infections compared with individuals who were naturally infected can be explained from two perspectives. Firstly, the higher reinfection rate among individuals with breakthrough infections can be attributed to differences in booster immunization patterns and the interval between the last booster dose and the initial infection. Although booster coverage was similar between the two groups, individuals who were naturally infected generally received more booster doses and had a longer interval between the last booster and infection. These factors may have contributed to their stronger and more sustained immune response, thereby reducing their reinfection risk. Secondly, we believe that individuals who experienced breakthrough infections may have had a weaker response to the vaccine because of genetic factors, health conditions, and other immune-related factors. As a result, despite receiving the COVID-19 vaccine, they were still susceptible to breakthrough infections and are more likely to experience reinfections in the future.

Our study found that as the number of booster vaccination doses increases after initial infection, the reinfection rate decreases. Considering the economic pressure of vaccination costs, receiving a single booster dose after infection can significantly reduce the risk of reinfection, which is consistent with existing research conclusions. Liang et al. demonstrated a significant increase in vaccine-induced NAb levels after Delta infection in vaccinated participants compared with unvaccinated persons, as in an earlier study [23]. Our study also found that as the interval between the last booster dose and the initial infection increased, there was a downward trend in the reinfection rate, although the intergroup difference was insignificant. This suggests that we can consider performing a booster immunization about one year after the initial infection, which may be more effective in preventing reinfection with new variant strains.

There was no significant statistical difference in the NAb titers during the recovery period after Delta strain infection between the individuals who experienced Omicron reinfection and those who did not experience reinfection, which were 9702.6 U/mL and 10,369.2 U/mL, respectively. Even among individuals without a history of vaccination, there was still no difference in neutralizing antibody levels between the two groups. In those individuals with the upper quartile of NAb titers during the recovery period after Delta strain infection, 16.9% also experienced Omicron reinfection. Moreover, in those individuals with the lower quartile of NAb titers, 84.1% did not experience Omicron reinfection. There was a lack of direct correlation between Delta NAb levels and reinfection with the Omicron BA.5 variant. From the analysis of NAb levels in both initial infections and reinfections in 28 individuals, we observed that the NAb levels during reinfection were not influenced by the levels during initial infection, regardless of their magnitude. Neutralizing antibody levels were more significantly affected by recent infections compared to earlier infections. Furthermore, it is important to consider the possibility that pre-existing neutralizing antibodies may contribute to an immunofacilitation phenomenon. This phenomenon can lead to enhanced viral entry into target cells, resulting in a higher viral load and more severe outcomes during reinfection with a different variant. This might explain part of the complexity of the immune responses observed in the reinfection scenarios. Additionally, we also performed cross-neutralization assays of Omicron BA.5 with sera from some individuals infected with the Delta strain. There was a 7.1-fold decrease in neutralizing ability against Omicron BA.5 in the serum, consistent with previous research [9,24].

### Limitations

While this study provides important insights, several limitations must be acknowledged. Firstly, we were unable to collect timely neutralizing antibody levels from individuals initially infected with the Delta strain prior to reinfection, which might have provided a clearer understanding of the reinfection process. Secondly, the sample size for the Omicron BA.5 cross-neutralization test was relatively small, resulting in a limited number of observed reinfections, and potentially a lack of representativeness when exploring factors that influence reinfection. Thirdly, subdividing the groups for stratified analysis might have led to insufficient statistical power in some subgroups, potentially increasing the risk of Type II errors. Further studies with larger sample sizes and more balanced group distributions are warranted to validate our results. Finally, our study only evaluated serum-neutralizing antibody levels and did not account for the role of cellular immunity, which could also play a significant role in the reinfection process.

## 5. Conclusions

Our study observed that neutralizing antibody levels in individuals previously infected with the Delta variant did not show a significant correlation with protection against subsequent reinfection with Omicron BA.5. This suggests that neutralizing antibodies generated from Delta infection alone may not provide substantial immunity against Omicron BA.5 reinfection. However, our study is limited by its small sample size and individual variability, which may have impacted the statistical power of our findings. Future studies with larger cohorts and more comprehensive analyses are needed to confirm these observations. Additionally, our results indicate that receiving a booster vaccination approximately one year after the initial infection could lower the risk of reinfection, though further investigation is necessary to validate this conclusion.

## Figures and Tables

**Table 1:** The incidence of Omicron BA.5 reinfection in individuals with a prior Delta infection.

Characteristics	Patients (n[%of Total])	Reinfection (n[%of Total])	Reinfection Rates (%)	*p ^a^*
Total	431 (100%)	77 (100%)	17.9%	
Age (mean ± SD)	50.1 ± 21.8	47.2 ± 19.3		0.270
Age (y)				0.011
<18	46 (10.7%)	5 (6.5%)	10.9%	
18~45	128 (29.7%)	33 (42.9%)	25.8%	
46~65	117 (27.1%)	23 (29.9%)	19.7%	
>65	140 (32.5%)	16 (20.8%)	11.4%	
Sex				0.174
Female	250 (58.0%)	50 (64.9%)	20.0%	
Male	181 (42.0%)	27 (35.1%)	14.9%	
Clinical classification at the time of initial infection *				0.598
Mild-type	65 (15.1%)	9 (11.7%)	13.8%	
Normal-type	346 (80.3%)	65 (84.4%)	18.8%	
Severe/critical-type	20 (4.6%)	3 (3.9%)	15.0%	
Breakthrough Infection Status				0.016
Yes	255 (59.2%)	55 (71.4%)	21.6%	
No	176 (40.8%)	22 (28.6%)	12.5%	
Booster immunization				0.007 ^b^
No	200 (46.4%)	49 (63.6%)	24.5%	
Yes (One dose)	112 (26.0%)	11 (14.3%)	9.8%	
Yes (Two doses)	89 (20.6%)	15 (19.5%)	16.9%	
Yes (Three doses or more)	30 (7.0%)	2 (2.6%)	6.7%	
Interval between the last booster dose and the initial infection (months)				0.626
3–6	38 (16.5%)	5 (17.9%)	13.2%	
6–9	61 (26.4%)	8 (28.6%)	13.1%	
9–12	47 (20.3%)	6 (21.4%)	12.8%	
>12	85 (36.8%)	9 (32.1%)	10.6%	

^a^ Chi-square test or Fisher’s exact test, as appropriate; ^b^ *p*-value was obtained from the Chi-square test for trends; * The clinical classification definitions refer to the “Diagnosis and Treatment Protocol for Novel Coronavirus Pneumonia (Trial version 7)”.

## Data Availability

The data are available from the authors upon reasonable request and with permission from the Jiangsu Provincial Center for Disease Control and Prevention.

## Supplementary Materials

The following supporting information can be downloaded at: https://www.mdpi.com/article/10.3390/vaccines12111211/s1, Figure S1: Box-whisker Plot of Delta NAb levels in Omicron BA.5 reinfection vs. Non-reinfection Individuals.; Table S1: Comparison of Booster Immunization Status and Time Interval Between the Last Booster Dose and Initial Infection in Naturally Infected and Breakthrough Infected Participants.; Table S2: The impact of immune status before initial infection on Omicron BA.5 reinfection in prior Delta-infected individuals.; Table S3: The effect of booster immunization after initial Delta infection on Omicron BA.5 reinfection.; Table S4: Omicron BA.5 reinfection and neutralizing antibody quartiles in Delta-infected individuals.; Table S5: Neutralizing Antibody (NAb) Levels Against Delta Variant Among 431 Study Participants.; Table S6: Neutralizing Antibody (NAb) Levels Against Delta and Omicron BA.5 Variants for 35 Samples Used in Heatmap Analysis.
