# Supplementary material for: Neutralizing Antibody-Mediated Protection from Prior Delta Variant Infection Against Omicron BA.5 Sub-Lineage Reinfection One Year Later: A Prospective Cohort Study
Source: Vaccines (Basel). 2024 Oct 25;12(11):1211. doi: 10.3390/vaccines12111211 (PMC11598881; doi:10.3390/vaccines12111211)

Suppl. Tables:

Table S1. Comparison of Booster Immunization Status and Time Interval Between the Last Booster Dose and Initial Infection in Naturally Infected and Breakthrough Infected Participants.

**Category of Population**	**Participants**	**Booster Immunization Status After Initial Infection**				**Booster coverage, %**	**P-value**	**Interval between the last booster dose and the initial infection, median (IQR)/months**
		**Non**	**one**	**two**	**three or more**			
**Naturally Infected**	**176**	**73**	**25**	**48**	**30**	**58.5**	**0.088**	**11.1 (8.6, 16.5)**
**Breakthrough Infected**	**255**	**127**	**87**	**41**	**0**	**50.2**		**8.9 (7.4, 15.3)**

Table S2. The impact of immune status before initial infection on Omicron BA.5 reinfection in prior Delta-infected individuals.

Initial infection	Whether reinfection	Samples (N)	GMC	IQR (Q25, Q75)	*P*
Natural infection	Yes	22	6588.3	(5266.3, 16005.1)	0.424
	No	154	6508.6	(2829.0, 16005.1)	
Breakthrough infection	Yes	55	11327.5	(6464.2, 32909.4)	0.336
	No	200	14841.7	(8272.8, 36567.0)	

Table S3. The effect of booster immunization after initial Delta infection on Omicron BA.5 reinfection.

Initial infection	Whether booster immunization	Whether reinfection	Samples (N)	GMC	IQR (Q25, Q75)	*P*
Natural infection	Yes	Yes	12	5559.2	(5658.1, 16005.1)	0.484
		No	91	8133.8	(4001.3, 16005.1)	
	No	Yes	10	8077.6	(1422.2, 16117.5)	0.211
		No	63	4716.9	(2001, 16005.1)	
Breakthrough infection	Yes	Yes	16	13018.7	(5044.7, 25213.6)	0.931
		No	112	13558.4	(9143.0, 36567.0)	
	No	Yes	39	10699.0	(6464.0, 36567.0)	0.229
		No	88	16652.1	(6464.2, 36567.0)	

Table S4. Omicron BA.5 reinfection and neutralizing antibody quartiles in Delta-infected individuals.

Nabs level	Reinfection (N, %)	Non-reinfection (N, %)	Total (N)	*P*
>Q75	20 (16.9%)	98 (83.1%)	118	0.834
<Q25	18 (15.9%)	95 (84.1%)	113	

Table S5. Neutralizing Antibody (NAb) Levels Against Delta Variant Among 431 Study Participants.

ID	Delta_NAb	ID	Delta_NAb	ID	Delta_NAb	ID	Delta_NAb
1	4001	2	93092	3	16005	4	16005
5	16005	6	16005	7	1422	8	16005
9	32909	10	5658	11	11316	12	16005
13	11316	14	16005	15	93092	16	32909
17	16005	18	16005	19	11316	20	16005
21	11316	22	5658	23	16005	24	11316
25	16005	26	16005	27	2829	28	4
29	8003	30	2001	31	16005	32	11316
33	16005	34	16005	35	16005	36	46546
37	4091	38	2829	39	16455	40	16005
41	16005	42	8273	43	11316	44	65819
45	5658	46	4091	47	5658	48	16005
49	703	50	2829	51	5658	52	5658
53	93092	54	16005	55	8003	56	2829
57	11316	58	5818	59	8273	60	2091
61	4091	62	16005	63	16005	64	32909
65	16005	66	23273	67	500	68	4
69	1422	70	16005	71	65819	72	1000
73	2829	74	2829	75	2091	76	4001
77	2829	78	16005	79	46546	80	46546
81	16005	82	16005	83	93092	84	1422
85	32909	86	46546	87	16005	88	4091
89	8003	90	11316	91	11316	92	23273
93	11316	94	16005	95	4091	96	65819
97	4	98	500	99	32909	100	1422
101	359	102	11316	103	11316	104	16005
105	16005	106	8003	107	2829	108	172
109	500	110	16005	111	16005	112	11316
113	11316	114	11636	115	5658	116	16005
117	2829	118	1422	119	703	120	16005
121	16005	122	2829	123	1422	124	2909
125	11636	126	5658	127	32909	128	4091
129	2909	130	703	131	4001	132	3232
133	2001	134	5818	135	5033	136	16005
137	359	138	2001	139	6464	140	16005
141	16005	142	2829	143	1000	144	5658
145	46546	146	65819	147	65819	148	32909
149	11316	150	2091	151	4091	152	1000
153	16005	154	11636	155	2091	156	2829

157	2829	158	2091	159	16005	160	32909
161	8003	162	1422	163	1422	164	16005
165	2001	166	8273	167	1422	168	4091
169	16455	170	1422	171	2001	172	1422
173	11316	174	16005	175	1000	176	8003
177	16455	178	32909	179	8273	180	11636
181	5818	182	32909	183	46546	184	18286
185	65819	186	4091	187	16455	188	16455
189	11636	190	8273	191	93092	192	16455
193	46546	194	32909	195	25860	196	32909
197	32909	198	4091	199	3233	200	16455
201	6464	202	25860	203	32909	204	8273
205	16455	206	32909	207	16455	208	32909
209	8273	210	8273	211	36572	212	32909
213	11636	214	32909	215	11636	216	46546
217	16455	218	8273	219	65819	220	32909
221	23273	222	65819	223	5818	224	8273
225	23273	226	16455	227	65819	228	93092
229	11636	230	18284	231	73144	232	51721
233	36572	234	73144	235	16005	236	73144
237	703	238	51721	239	103431	240	12928
241	16005	242	9142	243	36572	244	4
245	12930	246	4	247	12930	248	12930
249	25857	250	2286	251	36567	252	73144
253	12930	254	36567	255	6464	256	51721
257	4	258	12930	259	23273	260	36572
261	25860	262	11316	263	9143	264	9143
265	16005	266	11316	267	16005	268	16005
269	51715	270	11316	271	16005	272	16005
273	16005	274	8003	275	4001	276	16005
277	2829	278	73144	279	11316	280	16005
281	16005	282	16005	283	8003	284	36567
285	16005	286	4001	287	4	288	25860
289	4572	290	2285	291	3232	292	73144
293	12928	294	36567	295	73144	296	9143
297	73144	298	3233	299	6464	300	9143
301	18284	302	36567	303	46546	304	2001
305	32909	306	36572	307	73144	308	11316
309	4572	310	65819	311	1422	312	36567
313	16005	314	73144	315	16005	316	16005
317	12930	318	12928	319	12930	320	11636
321	25860	322	4	323	2091	324	6464
325	12930	326	3232	327	2285	328	4091

329	18284	330	2285	331	11316	332	18284
333	25857	334	3232	335	18286	336	1616
337	25860	338	6464	339	6464	340	6464
341	51721	342	73144	343	73144	344	6464
345	12928	346	16455	347	9143	348	51721
349	18286	350	16455	351	12930	352	6464
353	32909	354	11636	355	73144	356	3232
357	73144	358	73144	359	73144	360	73144
361	25857	362	36572	363	12930	364	6464
365	9143	366	1616	367	6464	368	6464
369	73144	370	4571	371	3232	372	36567
373	12928	374	8273	375	11636	376	12930
377	12928	378	36572	379	73144	380	12930
381	73144	382	73144	383	73144	384	25860
385	36567	386	2286	387	25860	388	32909
389	18284	390	3232	391	25860	392	18284
393	3232	394	73144	395	12928	396	4571
397	4091	398	16455	399	32909	400	36567
401	12928	402	4571	403	6464	404	25860
405	73144	406	73144	407	4	408	1143
409	12930	410	73144	411	16455	412	73144
413	6464	414	25860	415	46546	416	6464
417	6464	418	32909	419	36567	420	25857
421	32909	422	73144	423	51721	424	18286
425	73144	426	18286	427	36567	428	25857
429	16005	430	12928	431	73144		

Table S6. Neutralizing Antibody (NAb) Levels Against Delta and Omicron BA.5 Variants for 35 Samples Used in Heatmap Analysis.

ID	Delta_NAb	BA.5_NAb
Case 18	4	4
Case 30	4	4
Case 6	23	4
Case 23	32	32
Case 25	32	16
Case 15	45	16
Case 20	64	8
Case 4	91	4
Case 7	91	8
Case 14	91	8
Case 22	128	23
Case 2	181	8
Case 5	181	16

**Case 29**	**181**	**128**
**Case 31**	**181**	**16**
**Case 34**	**181**	**256**
**Case 35**	**256**	**16**
**Case 21**	**362**	**64**
**Case 24**	**362**	**256**
**Case 27**	**362**	**23**
**Case 32**	**362**	**16**
**Case 16**	**512**	**8**
**Case 26**	**512**	**64**
**Case 33**	**512**	**128**
**Case 1**	**724**	**11**
**Case 10**	**724**	**23**
**Case 19**	**724**	**181**
**Case 3**	**1024**	**16**
**Case 8**	**1024**	**91**
**Case 9**	**1024**	**11**
**Case 11**	**1024**	**91**
**Case 12**	**1024**	**91**
**Case 13**	**1024**	**32**
**Case 17**	**1024**	**128**
**Case 28**	**1024**	**362**

Suppl. Figure:

Figure S1: Box-whisker Plot of Delta NAb levels in Omicron BA.5 reinfection vs. Non-reinfection Individuals. The Y-axis was scaled in Log2 units. The blue and red circles represented individual data points, ranging from the minimum to the maximum values. NAb levels below the dashed line were considered negative. *P* value was determined by applying Wilcoxon Signed Rank Test.

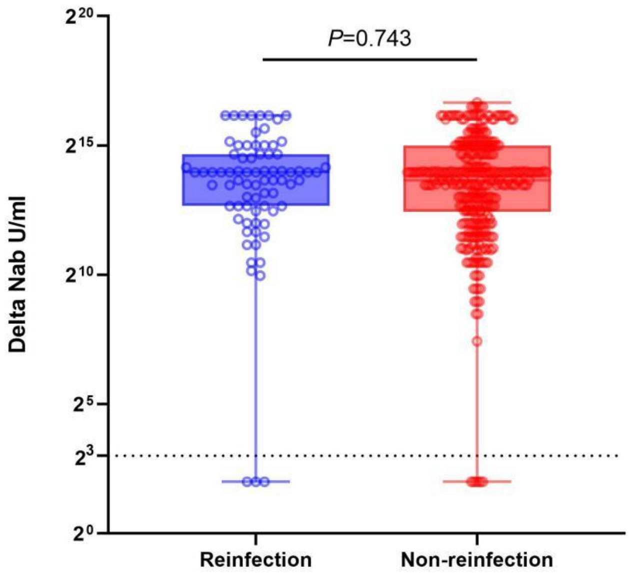

Supplement: Supplementary file 1 [file vaccines-12-01211-s001.zip › vaccines-3242541-supplementary.pdf]
